# Timely Birth Dose Vaccine to Prevent Vertical Transmission of Hepatitis B: A Single Center Experience on the Road to the WHO Elimination Goals in Italy

**DOI:** 10.3390/vaccines9070801

**Published:** 2021-07-19

**Authors:** Michele Pinon, Laura Giugliano, Emanuele Nicastro, Omar Kakaa, Alessandra Coscia, Caterina Carbonara, Lorenzo D’Antiga, Pier Luigi Calvo

**Affiliations:** 1 Pediatric Gastroenterology Unit, AOU Città della Salute e della Scienza di Torino, University of Turin, 10126 Turin, Italy; l.giugliano91@gmail.com (L.G.); pierluigi.calvo@unito.it (P.L.C.); 2 Paediatric Hepatology, Gastroenterology and Transplantation, ASST Ospedale Papa Giovanni XXIII, 24127 Bergamo, Italy; enicastro@asst-pg23.it (E.N.); ldantiga@asst-pg23.it (L.D.); 3 Department of Pediatrics and Public Health Sciences, University of Turin, 10126 Turin, Italy; omar.kakaa@unito.it; 4 Neonatology Unit, AOU Città della Salute e della Scienza di Torino, University of Turin, 10126 Turin, Italy; alessandra.coscia@unito.it (A.C.); ccarbonara@cittadellasalute.to.it (C.C.)

**Keywords:** hepatitis B virus, vertical transmission, timely vaccination, immunoprophylaxis, prevention strategies

## Abstract

Italy was one of the first industrialized countries to implement a program of routine vaccination against hepatitis B virus (HBV) infection. However, currently, no HBV vaccine is administered at birth if the screened mother is HBsAg negative, whilst babies born to HBsAg positive mothers are given vaccine and hepatitis B immunoglobulin, within 12–24 post-delivery hours. A single center retrospective analysis of policies and practices to prevent mother-to-child transmission of HBV was carried out, to evaluate their adherence to HBV care guidelines. Paired maternal-infant medical records for consecutive live births, between January 2015 and December 2019, were reviewed at the AOU Città della Salute e Scienza di Torino, where a total of 235/35,506 babies (0.7%) were born to HBsAg positive mothers. Markers of active viral replication, i.e., HBV DNA level and/or HBeAg, were reported in only 66/235 (28%) of the mothers’ medical records. All newborns had immunoprophylaxis at birth: 61% at <12 h, 31% between 12 and 24 h, 7% between 24 and 36 h and 1% at >36 h. In 2019, two cases of vertical HBV transmission occurred, despite timely immunoprophylaxis, as their mothers’ viral load was detected too late for antiviral prophylaxis. Missed early identification of pregnant women with high viremia levels or late vaccinations may contribute to perinatal HBV infection. Immunoprophylaxis should be given to babies born to HBsAg positive mothers at the latest within 12 h. In Italy, policies aimed at achieving the WHO 2030 goal of eliminating viral hepatitis should be further implemented.

## 1. Introduction

In 1991, Italy was one of the first industrialized countries to implement routine vaccination against hepatitis B virus (HBV) infection. It involved universal vaccination of all infants with a 3-dose series during the first year of life (at 3, 5, and 11 months of age) and of all 12-year-old children during the first 12 years of the program [1,2,3,4]. The screening of pregnant women and subsequent administration of HBV vaccine and hepatitis B immunoglobulin (HBIG) at birth, within 12–24 h from delivery, to newborns from HBsAg positive mothers was also adopted [5,6]. This vaccination program had a positive clinical and economic impact during the first 30 years of adoption, leading to an extensive decrease in disease burden, HBsAg carrier rates, and hepatitis B-related morbidity and mortality in the Italian population. After obtaining a substantial improvement in HBV prevalence over the last few decades, Italy currently has a low endemicity [7]. According to the Acute Hepatitis National Surveillance System (SEIEVA), the incidence of acute hepatitis B decreased from 12 per 100,000 inhabitants in 1985, to 1.0 per 100,000 in 2011, dropping to 0.4 per 100,000 in 2019 [8]. The HBsAg positive rate in the general population is estimated to be between 0.8 and 1% [9]. An a posteriori economic analysis evidenced the positive impact of the adoption of universal vaccination against hepatitis B in Italy from 1991 to 2010 [10]. It is expected that there will be further clinical and economic benefits from this first vaccination period in the future, due to a foreseen reduction in chronic hepatitis B, cirrhosis, hepatocellular carcinoma cases, and, consequently, in costs related to care/treatment [10,11].

The World Health Organization (WHO) emphasizes that prevention of vertical transmission of hepatitis B is pivotal in reducing HBV incidence and eliminating viral hepatitis by 2030. [12]. However, the deadline is fast approaching, and although progress has been made, some important gaps, in terms of public health policies, must be filled in before this goal can be reached. The WHO has always recommended the scaling up of hepatitis B vaccine birth dose coverage, making global reduction in HBV infection more likely [13]. As the risk of chronic HBV infection is highest when it is acquired at birth, it has been estimated that there would be a 16% reduction in the global burden of chronic HBV infection, if the vaccine birth dose were routinely given to prevent vertical hepatitis B transmission. Moreover, it has been postulated that the implementation of the vaccine birth dose could avoid a potential 1.2–1.5 million HBV infections and 0.3–1.2 million vertical infection-related deaths from 2021–2025 [14,15]. Nevertheless, worldwide estimations indicate that only 39% of newborns are vaccinated at birth. Many African countries are not able to administer the vaccine within the recommended time window, and, therefore, it is not part of their national policies. Currently, Italy is one of the high-income countries where cost-benefit analysis induces healthcare authorities not to vaccinate newborns at birth if the screened mother is HBsAg negative [16].

A retrospective analysis of the policies and practices implemented to prevent HBV vertical transmission was carried out in the Italian hospital with the highest number of deliveries per year. This aimed at obtaining data on the prevalence of HBsAg positivity in pregnant women and estimating the level of adherence our policies and practices had to

HBV care guidelines. The barriers to reaching the WHO goal of eliminating viral hepatitis by 2030 in a high-income country with a low HBV endemicity were assessed and potential strategies, aimed at optimizing the current care pathways, were also investigated.

## 2. Materials and Methods

This single-center retrospective study was based on a revision of the Electronic Health Records (EHR) of pregnant women who had given birth between January 2015 and December 2019 at the AOU Città della Salute e Scienza di Torino, Italy. All consecutive HBsAg-positive pregnant women were selected according to the following criteria: (i) HBsAg positivity during pregnancy or at birth, (ii) delivery between 1st January 2015 and 31st December 2019. The first reported pregnancy was analyzed for eligible women with more than one pregnancy during the study period. Infants of mothers with HBV were identified through the linkage of maternal and infant charts in the EHR. A dedicated database was used to make retrospective data collection from clinical records and digital archives. The following data were extracted from paired maternal-infant medical records of HBsAg positive women and their babies:

- Mothers’ age, country of origin, parity, and mode of birth;
- maternal HBsAg status at birth (assessed by biochemical luminescence microparticle immunoassay, CMIA);
- the laboratory data included HBV DNA level during pregnancy and other serologic markers of HBV maternal infection (e.g., HBcAb total, HBcIgM, HBeAg, HBeAb), when available;
- the maternal HIV and HCV co-infection status;
- the newborns’ gender, birth weight, gestational age at birth, and feeding method.

Adherence to the prevention strategies adopted before, at, and after birth was recorded, e.g., referral of HBsAg pregnant women to an HBV specialist, HBV DNA testing and starting antiviral prophylaxis when indicated (i.e., high viral load infection), the administration of immunoprophylaxis at birth and its timing from birth. However, adherence to post-vaccine serologic testing could not be evaluated as it was not provided by our center at the time of the study.

Maternal and child delivery factors associated with the timely administration of the infant immunoprophylaxis (hepatitis B vaccine and HBIG) within 12 h of birth were also evaluated. Maternal high viral load infection was defined as an HBV DNA of >200,000 IU/mL or HBeAg positivity. Immunoprophylaxis was defined as the administration of HBV vaccine and HBIG to babies born to HBsAg-positive women. HBV vertical transmission was defined as positivity at 6–12 months of life of HBsAg or HBV DNA in an infant born to an infected mother.

Statistical analysis: categorical variables were summarized as counts (*n*) and proportions (%). Logistic regression analyses were performed to test the predictive value of any variables that had a statistically significant correlation with immunoprophylaxis timeliness. All the tests were two-tailed and a *p*-value of ≤0.05 was considered statistically significant. The confidence intervals (CI) were calculated at the 95% level.

The data were analyzed by the RStudio Version 1.4.1106 software package. The study was approved by the Institutional Review Board (Comitato Etico Interaziendale AOU Città della Salute e della Scienza di Torino, Italy; IRB number: 073.630). The study was carried out according to the Declaration of Helsinki and the Good Clinical Practice guidelines.

## 3. Results

A total of 235/35,506 (0.7%) babies were born to 231 HBsAg-positive mothers. There were four sets of twin siblings included in the cohort. Only 31/235 (13.2%) of the babies were born to Italian mothers, 140 (59.9%) were from Eastern Europe (i.e., Romania, Albania, and Moldova), 29 (12.3%) from Asia, and 35 (14.9%) were of African origin. All the HBsAg positive Italian women were >25 years of age, whereas most of the HBsAg positive women from other countries (151/201, 75.1%) were <35 years old.

The baseline characteristics of the HBsAg-positive women and their babies, stratified by their Italian or foreign origin, are reported in Table 1.

**Figure 1:**
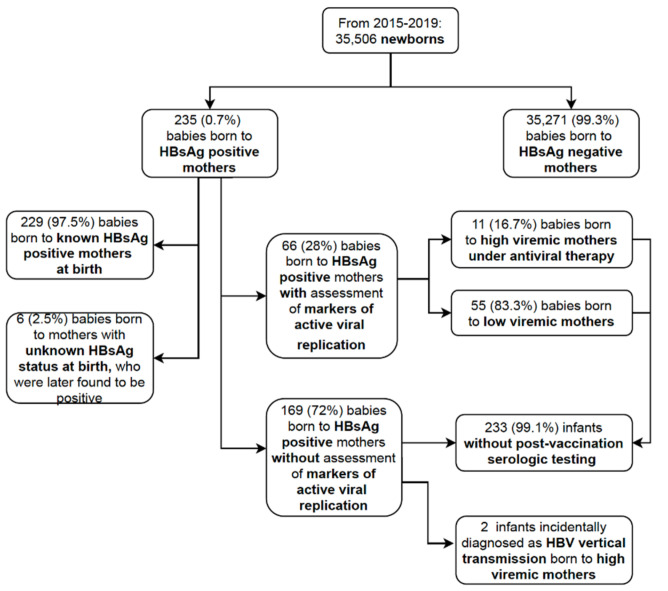
Flowchart outlining the study population selection.

All pregnant women were tested for HBsAg either during pregnancy or at admission for childbirth. Nevertheless, exactly when maternal HBsAg screening was performed during pregnancy could not be determined as it had not been noted in the mothers’ medical records. Maternal HBsAg status was known at birth in 229/235 (97.5%) babies born to HBsAg positive women. Only 6/235 (2.5%) of the babies were born to women with unknown serologic status at birth: these women were tested at admission and were found to be HBsAg positive only after having given birth (Figure 1).

Information as to markers of active viral replication, i.e., HBV DNA level and/or HBeAg, was retrievable in only 66/235 (28%) of the mothers’ medical records: 11 (16.7%) had a high viral load infection (8/11 from foreign countries: 3 from Asia, 3 from Eastern Europe, 2 from Africa), whereas 55 (83.3%) had low viremia levels. Seven out of the 11 high viremic women had timely detection of their high viral load. Therefore, antiviral prophylaxis (tenofovir in 6, lamivudine in 1) was started during the third trimester of pregnancy. Tenofovir had been administered before pregnancy to the remaining 4/11 women with chronic-active hepatitis B (Figure 1). As no mention had been made of other HBV maternal infection serologic markers, it was not possible to classify the remaining 169 (72%) HBsAg positive women according to high or low viral load infection.

As to co-infection, 231/235 (98.3%) women were tested for HIV (2 were positive) and only 95 (40.4%) for HCV (all negative).

**Figure 2:**
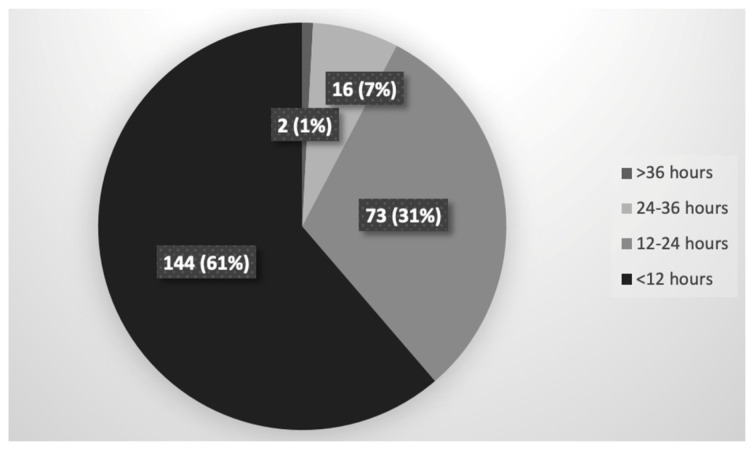
Timing of immunoprophylaxis (vaccine + HIBG at the same time).

All newborns were given immunoprophylaxis (vaccine + HIBG at the same time): 144/235 (61%) at <12 h from birth, 73/235 (31%) at 12–24 h, 16/235 (7%) at 24–36 h and 2/235 (1%) at >36 h from birth (Figure 2).

Attention was then focused on the factors affecting immunoprophylaxis timeliness (Table 2). Maternal parity and birth time were the only statistically significant predictive factors associated with timely immunoprophylaxis (<12 h from birth): babies born to multiparous women or those born during the day were given HBV vaccine and HBIG earlier than those born to primiparous women (*p*-value = 0.01) or during the night (*p*-value = 0.03), respectively.

The rate of vertical HBV transmission was not evaluated as no post-vaccination serologic testing had been carried out before the study was concluded. Nevertheless, two infants were referred to our center due to the incidental finding of slightly elevated transaminases levels and, despite timely immunoprophylaxis, vertical transmission of HBV was diagnosed. Their mothers’ high viral load was not detected in time for antiviral prophylaxis during pregnancy (Table 3).

## 4. Discussion

The data from the Italian HBV vaccination program emphasize the positive medium-long-term role universal immunization plays in HBV eradication and justify investments in prevention. Universal vaccination with the screening of pregnant women, along with timely immunoprophylaxis at birth, remains the most effective strategy to reduce HBV infection, if and when the financial condition of a country allows for the adoption of such a program. Indeed, it has been reported that the administration of HBIG leads to as high as a 95% increase in the prevention rate of perinatal transmission [17,18,19,20].

Nevertheless, our analysis did bring to light some critical issues in the current Italian hospital policies, including an incomplete adherence to international recommendations. Indeed, although Italy and some other countries do have a successful prevention program and are close to achieving the WHO elimination target, it is advisable to evaluate the potential impact of a further scale-up.

In Italy, immunoprophylaxis is provided to babies born to HBsAg positive mothers within a maximum of 24 h from birth, even if the AASLD (American Association for the Study of Liver Diseases), CDC (Centers for Disease Control and Prevention)/ACIP (Advisory Committee on Immunization Practices) and the EASL (European Association for the Study the Liver) guidelines recommend it be done within 12 h [1,2,3,21,22,23]. Our data showed that 100% of newborns had immunoprophylaxis within the timeframe proposed by the Italian guidelines in 92.3% of cases [5,6]. Nevertheless, only in 61.3% of cases was it administered within the 12 h set by the main international guidelines [1,2,3,21,22,23]. Furthermore, early vaccination has proven to be more effective. Indeed, Jourdain et al. [24] observed that vertical transmission was notably low (only 2%) in women with high viremia levels when the HBIG and the vaccine birth doses were administered early (median 1.3 and 1.2 h, respectively). Nevertheless, antiviral prophylaxis during pregnancy in high viremic HBsAg-positive mothers still remains pivotal, pending further research.

The prevention of hepatitis B vertical transmission depends also on full adherence to the recommended policies. The CDCs report approximately 40–90 perinatal infections/year in the USA, which may even be 10 to 20-fold underestimated [25]. Willis et al. observed inadequate immunoprophylaxis in 242 birth centers in the USA for infants born to HBsAg positive mothers and women with unknown serologic status, whilst it was carried out correctly in 62.1% and 52.4% of cases, respectively [25,26]. Denmark reported 4.0% of missed HBIG administration at birth, Italy 5.0%, the USA 19.7%, and China 62.4% [26]. The correct timing of the vaccine birth dose is crucial to reduce the infection risk, as it has been proven that efficacy is reduced if delayed beyond 24 h after birth [27,28]. Delayed 3-dose series vaccinations were not only observed in low-income, but also in high-income countries [29,30,31,32]. Indeed, the late vaccinations we observed in 7.7% of our cases at >24 h from birth (6.8% within 24–36 h, 0.9% > 36 h) might lead to perinatal HBV infection.

Numerous maternal and delivery factors may contribute to immunoprophylaxis delay. The late immunoprophylaxis (>12 h from birth) we observed in babies born to primiparous HBsAg positive women may depend on the physician and mother having poor awareness of the risk of vertical transmission in this group. Conversely, immunoprophylaxis was administered earlier in babies born to multiparous women, who probably had managed their HBsAg positive status during a previous pregnancy. Moreover, the late immunoprophylaxis in babies delivered during the night emphasizes the need for care pathways able to provide timely HBV vaccines and HBIG also at night. As early administration had proven to be efficacious [24], further strategies should be developed to provide better immunoprophylaxis timeliness. One option may be that of obtaining consent from HBsAg positive mothers at admission for childbirth, to be able to administer HBV vaccines and HBIG to their babies immediately after birth in the delivery room, also.

The results obtained in our study were in line with the rate of HBsAg positive subjects in the general Italian population, i.e., a 0.7% prevalence of HBsAg positive pregnant women, confirming that most of the HBsAg positive pregnant women were not of Italian origin. A recent multicenter study by the Italian Association of Infectious and Tropical Diseases [33] on 3760 HBsAg positive subjects reported that 24.8% were immigrants and emphasized the importance of screening this population. In 2021, although HBV infection may occur in Italian non-vaccinated subjects over 42 or vaccine non-responders younger women, it mainly affects people born outside Italy in countries with high or intermediate HBV endemicity, which make up about 8.5% of the Italian population [34,35]. Moreover, the age groups differed, as most of the HBsAg-positive women from foreign countries were <35 years old.

As there is currently no universal vaccine birth dose program in Italy, infants born to HBsAg negative mothers from high endemic countries are at risk of horizontal infection during the period preceding the vaccine series [36]. If the birth dose is not administered on a large scale, then also household contacts from these high endemic countries should be tested and vaccinated. Indeed, after having carried out a detailed cost-benefit analysis, another option for healthcare authorities could be that of extending the vaccine birth dose to babies born to HBsAg negative mothers from these countries.

Newborns from women with unknown HBsAg status may not be vaccinated in time if the vaccine birth dose is not widely administered. When the maternal serologic status is unknown, international guidelines recommend HBsAg testing be carried out as soon as possible. Should the result not arrive in due time, the newborn should be vaccinated within 12 h of birth, particularly if their mothers come from highly endemic countries, even if they are young women. The administration of specific HBIG is also recommended should the mother be HBsAg positive [1,2,3,21,22,23]. Unfortunately, Italy does not yet have a clearly defined prevention strategy, which risks delaying immunoprophylaxis beyond the recommended time window. Luckily, our results showed that the serologic status at delivery was available for most of our HBsAg-positive mothers (97.5%), ensuring immunoprophylaxis to their newborns without delay.

However, inappropriate or late vaccine schedules may not be the only factors involved in vaccine failure. Indeed, the most significant factor for vertical HBV transmission is the level of maternal HBV DNA at birth: 10–20% of infants born to mothers with high viremia levels still acquire HBV, despite correct immunoprophylaxis [2,3]. As of 2015, the AASLD recommends testing maternal HBsAg status as soon as pregnancy is confirmed and advocates the use of antiviral prophylaxis during the third trimester in women with a high viral load [3,23]. Although this is considered a rare observation in Italian pregnant women, it was observed in 16.7% of the women tested in our population, who were mainly from countries other than Italy. The scarcity of timely identification of high viremia levels and early antiviral prophylaxis in pregnancy might also contribute to perinatal HBV infection, despite timely immunoprophylaxis, as observed in our two cases of vertical HBV transmission.

Our study demonstrated that there is not always appropriate surveillance of HBsAg-positive women during pregnancy and evidenced that only rarely were markers of active viral replication, i.e., HBV DNA level or HBeAg, reported in the mothers’ medical records. This gap may reflect limited awareness of HBV care during pregnancy and inadequate communication between obstetricians and HBV specialists. Kushner et al. observed that providing HBV care in obstetrics departments improved adherence to maternal care measures, i.e., referral to an HBV specialist, HBV DNA testing, and starting of antiviral prophylaxis when indicated [32]. We think that increasing connection between all the healthcare providers involved in the care pathways to prevent mother-to-child transmission of HBV, i.e., obstetricians, neonatologists, and HBV specialists, may improve the screening and care of HBsAg positive pregnant women and, consequently, future maternal and infant health.

Furthermore, according to international guidelines, post-vaccination serologic testing for infants born to HBsAg positive mothers should be carried out to identify infected subjects or vaccine non-responders, who, as they are at risk of horizontal infection, should be revaccinated. A further question remains that of the identification of hepatitis B occult carriers (HBsAg−/HBcAb+), observed in 3.3% of cases in Italy [37], among infants born to HBsAg positive mothers. As anti-HBc antibodies may be missed by the post-vaccination serologic monitoring at 9–12 months [29], it might be extended up to 18–24 months of life. We think that determining anti-HBc antibodies in infants born to HBsAg positive mothers is of paramount importance, as HBV reactivation concomitant to immunosuppression in hepatitis B occult carriers is a recognized clinical problem on the increase [38,39].

The fact that our study evidenced that less than 50% of HBsAg-positive women were tested for HCV is alarming, considering that the co-existence of infection risk factors common to both viral infections is frequent. Currently, in Italy, HCV screening is recommended for women at risk of contracting the infection and it is often not implemented, even if AASLD guidelines recommend that all pregnant women should be tested for HCV infection [40].

This study reports a preliminary, single center analysis. We are aware that it does have some limitations, including the fact that most of the mothers’ medical records were incomplete and it was not possible to evaluate some hospital policies and practices. Moreover, no data were available on post-vaccination serologic monitoring, as a clinical and laboratory follow-up for infants born to HBsAg positive mothers has only recently been set up in our center. Although these shortcomings could be a limitation of the study, we think that they strengthen the argument in favor of the need for an implementation of HBV prevention strategies.

## 5. Conclusions

The recent advent of other global epidemics, polarizing the interest and consuming resources of public health services, may lead to viral hepatitis being even more neglected than before, risking putting aside the goal of HBV elimination.

We believe that the vaccine strategies in Italy should be optimized as they are the most cost-effective way of controlling HBV infection. Immunoprophylaxis should be given to babies born to HBsAg positive mothers within a maximum of 12 h, and even earlier immunoprophylaxis (within 4 h from birth) might be encouraged [24]. If large-scale vaccine birth doses cannot be implemented, further strategies should be developed to better protect babies born to HBsAg negative mothers or women with unknown serologic HBsAg status.

Moreover, the significant impact the Italian vaccination program had might have led to a false perception about the efficacy of immunoprophylaxis, by not taking into consideration the possibility of vaccine failure. Any prevention program should include an early full serologic HBV assessment should the mother be HBsAg positive, to timely identify pregnant women with high viral load infection and treat them as soon as possible, as well as a structured post-vaccination serologic testing of their infants. We think that there is a need to implement a multidisciplinary clinical pathway with a stronger connection between the prenatal, perinatal, and postnatal phases, as from the first stages of pregnancy to the second year of the child’s life, involving several healthcare providers. Moreover, closing and/or filling in the gaps between the recommended protocol and routine practices is pivotal.

In conclusion, further effort should be made to reach the WHO 2030 goal of eliminating viral hepatitis as a public health threat.

## Figures and Tables

**Table 1:** Baseline characteristics of HBsAg positive women and their infants. Abbreviations: Kg = kilograms; mixed feeding = breastfeeding combined with bottle feeding.

Variable	Italian Origin *N* (%)	Foreign Origin *N* (%)	Total *N* (%)
**Mothers’ Age**			
<25 years	0	15 (7)	15 (6)
25–35 years	13 (42)	138 (68)	151 (64)
>35 years	18 (58)	51 (25)	69 (30)
**Parity**			
Primiparous	13 (42)	74 (36)	87 (37)
Multiparous	18 (58)	130 (64)	148 (63)
**Mode of Birth**			
Natural birth	24 (77)	134 (66)	158 (67)
Scheduled Cesarean	3 (10)	38 (18)	41 (18)
Urgent Cesarean	4 (13)	32 (16)	36 (15)
**Gestational Age at Birth**			
<37 weeks	4 (13)	15 (7)	19 (8)
≥37 weeks	27 (87)	189 (93)	216 (92)
**Markers of Active Viral Replication (** **HBV DNA** **and/or HBeAg)**			
Reported in medical records	12 (39)	54 (26)	66 (28)
Not reported in medical records	19 (61)	150 (74)	169 (72)
High viremic women	3 (25)	8 (15)	11 (17)
Low viremic women	9 (75)	46 (85)	55 (83)
HBV DNA levels (IU/mL) in high viremic women; median (range)	200,001 (200,001–143,000,000)	785,000 (350,000–170,000.000)	460,573 (200,001–170,000,000)
Median HBV DNA levels (IU/mL) in low viremic women; median (range)	28 (<20–6000)	22 (<20–22,228)	22 (<20–22,228)
**Newborns’ Gender**			
Male	15 (48)	109 (53)	124 (53)
Female	16 (52)	95 (47)	111 (47)
**Birth Weight**			
<2.5 Kg	4 (13)	16 (8)	20 (9)
≥2.5 Kg	27 (87)	188 (92)	215 (91)
**Feeding Method**			
Breastfeeding	20 (65)	107 (52)	127 (54)
Bottle feeding	1 (3)	48 (24)	49 (21)
Mixed feeding	10 (32)	49 (24)	59 (25)
**Day of the Week for Birth**			
On a weekday	21 (68)	156 (76)	177 (75)
At weekends	10 (32)	48 (24)	58 (25)
**Birth Time**			
Day-time	11 (35)	83 (41)	94 (40)
Night-time	20 (65)	121 (59)	141 (60)

**Table 2:** Risk factors for late immunoprophylaxis at birth (>12 h from birth) in babies born to HBsAg positive mothers. Abbreviations: OR = Odds Ratio; CI = Confidence Intervals; g = grams. * significant risk factors (*p*-value ≤ 0.05).

	Late Immunoprophylaxis Proportion (%)	OR (95% CI)	*p*-Value
**Mothers’ Age**			
<25 years	1/15 (7)	1 (ref)	-
25–35 years	35/151 (23)	4.34 (0.55–34.17)	0.16
>35 years	10/69 (14)	2.99 (0.35–25.04)	0.31
**Country of Origin**			
Italy	7/31 (23)	1 (ref)	
Foreign country	42/204 (21)	0.89 (0.36–2.20)	0.80
**Parity**			
Primiparous	29/87 (33)	1 (ref)	
Multiparous	20/148 (14)	0.43 (0.27–0.83)	0.01 *
**Gestational Age at Birth**			
<37 weeks	4/19 (21)	1 (ref)	
>37 weeks	45/216 (21)	0.99 (0.31–3.12)	0.98
**Markers of Active Viral Replication (** **HBV DNA** **and/or HBeAg)**			
Not reported in medical records	41/169 (24)	1 (ref)	
Reported in medical records	16/66 (24)	0.93 (0.46–1.89)	0.84
**Mode of Birth**			
Scheduled Cesarean	9/41 (22)	1 (ref)	
Natural birth	31/158 (20)	0.87 (0.38–2.00)	0.74
Urgent Cesarean	9/36 (25)	1.18 (0.41–3.41)	0.75
**Newborns’ Gender**			
Female	18/111 (16)	1 (ref)	
Male	31/124 (25)	1.72 (0.9–3.29)	0.10
**Birth Weight**			
<2500 g	6/20 (30)	1 (ref)	
>2500 g	43/215 (20)	0.72 (0.24–2.1)	0.54
**Feeding Method**			
Bottle feeding	6/49 (12)	1 (ref)	
Breastfeeding	28/127 (22)	1.73 (0.70–4.28)	0.46
Mixed feeding	15/59 (25)	1.95 (0.72–5.31)	0.51
**Day of the Week for Birth**			
On a weekday	36/177 (20)	1 (ref)	
At weekends	13/58 (22)	1.13 (0.55–2.32)	0.37
**Birth Time**			
Day-time	13/94 (14)	1 (ref)	
Night-time	36/141 (26)	2.13 (1.06–4.29)	0.03 *

**Table 3:** Characteristics of two cases of vertical HBV infection. Abbreviations: HBV DNA = hepatitis B virus deoxyribonucleic acid; IU = international unit; + = positive; HBsAg = hepatitis B surface antigen; HBeAg = hepatitis B e antigen; HBsAb = antibody to hepatitis B surface antigen; HBcAb = antibody to hepatitis B core antigen; s/co: signal/cut-off; AST = alanine aminotransferase; ALT = aspartate aminotransferase.

	Case 1	Case 2
Post-partum maternal viral load	>170,000,000 UI/mL	>170,000,000 UI/mL
Maternal antiviral prophylaxis	no	no
Immunoprophylaxis (HBV vaccine + HIBG) at birth	<6 h from birth	<6 h from birth
Scheduled vaccination	regular	regular
Age at diagnosis	12 months	18 months
Child HBV DNA (IU/mL)	>170,000,000	>170,000,000
Child HBsAg (s/co)	+	+
Child HBeAg (s/co)	+	+
Child HBsAb (UI/mL)	<10	<10
Child HBcAb (s/co)	+	+
Child AST/ALT (UI/mL)	140/158	65/80

## References

[1] Weinbaum C.M., Williams I., Mast E.E., Wang S.A., Finelli L., Wasley A., Neitzel S.M., Ward J.W. (2008). Recommendations for identification and public health management of persons with chronic hepatitis B virus infection. MMWR Recomm. Rep..

[2] Schillie S., Vellozzi C., Reingold A., Harris A., Haber P., Ward J.W., Nelson N.P. (2018). Prevention of Hepatitis B Virus Infection in the United States: Recommendations of the Advisory Committee on Immunization Practices. MMWR Recomm. Rep..

[3] Terrault N.A., Lok A.S.F., McMahon B.J., Chang K.M., Hwang J.P., Jonas M.M., Brown R.S., Bzowej N.H., Wong J.B. (2018). Update on Prevention, Diagnosis, and Treatment of Chronic Hepatitis B: AASLD 2018 Hepatitis B Guidance. Clin. Liver Dis..

[4] LEGGE 27 maggio 1991, n. 165 Obbligatorieta’ della Vaccinazione Contro L’epatite Virale B. (GU Serie Generale n.127 del 01-06-1991). https://www.gazzettaufficiale.it/eli/id/1991/06/01/091G0201/sg#:~:text=note%3A%20Entrata%20in%20vigore%20della%20legge%3A%2016%2F6%2F1991&text=1.-,1.,nel%20primo%20anno%20di%20vita.

[5] Linea Guida Gravidanza Fisiologica (2011). Sistema Nazionale per le linee Guida Istituto Superiore di Sanità (SNLG-ISS), Aggiornamento. http://www.salute.gov.it/imgs/C_17_pubblicazioni_1436_allegato.pdf.

[6] Piano Nazionale Prevenzione Vaccinale PNPV 2017-2019. G.U. Serie Generale, n. 41 del 18 Febbraio 2017. https://www.gazzettaufficiale.it/eli/gu/2017/02/18/41/sg/pdf.

[7] Campagna M., Siddu A., Meloni A., Murru C., Masia G., Coppola R.C. (2011). Epidemiological impact of mandatory vaccination against hepatitis B in Italian young adults. Hepat. Mon..

[8] Sistema Epidemiologico Integrato dell’Epatite Virale Acuta (SEIEVA) Istituto Superiore di Sanità. Bollettino Annuale 2019. http://www.iss.it/seieva.

[9] (2015). 2015 Assessment Report of the Global Vaccine Action Plan.

[10] Boccalini S., Taddei C., Ceccherini V., Bechini A., Levi M., Bartolozzi D., Bonanni P. (2013). Economic analysis of the first 20 years of universal hepatitis B vaccination program in Italy: An a posteriori evaluation and forecast of future benefits. Hum. Vaccines Immunother..

[11] Lai A., Sagnelli C., Presti A.L., Cella E., Angeletti S., Spoto S., Costantino S., Sagnelli E., Ciccozzi M. (2018). What is changed in HBV molecular epidemiology in Italy?. J. Med. Virol..

[12] (2020). Prevention of Mother-to-Child Transmission of Hepatitis B Virus: Guidelines on Antiviral Prophylaxis in Pregnancy.

[13] Cornberg M. (2018). Annex C: Hepatitis B Birth dose Investment Case. GAVI.

[14] Goldstein S.T., Zhou F., Hadler S.C., Bell B.P., Mast E.E., Margolis H.S. (2005). A mathematical model to estimate global hepatitis B disease burden and vaccination impact. Int. J. Epidemiol..

[15] de Villiers M.J., Gamkrelidze I., Hallett T.B., Nayagam S., Razavi H., Razavi-Shearer D. (2020). Modelling hepatitis B virus infection and impact of timely birth dose vaccine: A comparison of two simulation models. PLoS ONE.

[16] (2017). Global Hepatitis Report.

[17] Wong V.C., Ip H.M., Reesink H.W., Lelie P.N., Reerink-Brongers E.E., Yeung C.Y., Ma H.K. (1984). Prevention of the HBsAg carrier state in newborn infants of mothers who are chronic carriers of HBsAg and HBeAg by administration of hepatitis-B vaccine and hepatitis-B immunoglobulin. Double-blind randomised placebo-controlled study. Lancet.

[18] Lo K.J., Tsai Y.T., Lee S.D., Wu T.C., Wang J.Y., Chen G.H., Yeh C.L., Chiang B.N., Yeh S.H., Goudeau A. (1985). Immunoprophylaxis of infection with hepatitis B virus in infants born to hepatitis B surface antigen-positive carrier mothers. J. Infect. Dis..

[19] Beasley R.P. (2009). Rocks along the road to the control of HBV and HCC. Ann. Epidemiol..

[20] Indolfi G., Easterbrook P., Dusheiko G., Siberry G., Chang M.H., Thorne C., Bulterys M., Chan P.L., El-Sayed M.H., Giaquinto C. (2019). Hepatitis B virus infection in children and adolescents. Lancet Gastroenterol. Hepatol..

[21] (2015). Guidelines for the Prevention, Care and Treatment of Persons with Chronic Hepatitis B Infection.

[22] European Association for the Study of the Liver (2017). EASL 2017 Clinical Practice Guidelines on the management of hepatitis B virus infection. J. Hepatol..

[23] Terrault N.A., Bzowej N.H., Chang K.M., Hwang J.P., Jonas M.M., Murad M.H., Diseases A.A. (2016). AASLD guidelines for treatment of chronic hepatitis B. Hepatology.

[24] Jourdain G., Ngo-Giang-Huong N., Harrison L., Decker L., Khamduang W., Tierney C., Salvadori N., Cressey T.R., Sirirungsi W., Achalapong J. (2018). Tenofovir versus Placebo to Prevent Perinatal Transmission of Hepatitis B. N. Engl. J. Med..

[25] Willis B.C., Wortley P., Wang S.A., Jacques-Carroll L., Zhang F. (2010). Gaps in hospital policies and practices to prevent perinatal transmission of hepatitis B virus. Pediatrics.

[26] Harder K.M., Cowan S., Eriksen M.B., Krarup H.B., Christensen P.B. (2011). Universal screening for hepatitis B among pregnant women led to 96% vaccination coverage among newborns of HBsAg positive mothers in Denmark. Vaccine.

[27] Wang C., Jia Z.F., Wu X., Wen S.M., Kong F., Hu K.Q., Li J., Jiang J., Niu J.Q. (2016). Protective effect of an improved immunization practice of mother-to-infant transmission of hepatitis B virus and risk factors associated with immunoprophylaxis failure. Medicine.

[28] Kang W., Ding Z., Shen L., Zhao Z., Huang G., Zhang J., Xiong Q., Zhang S., Wang F. (2014). Risk factors associated with immunoprophylaxis failure against mother to child transmission of hepatitis B virus and hepatitis B vaccination status in Yunnan province, China. Vaccine.

[29] Komatsu H. (2014). Hepatitis B virus: Where do we stand and what is the next step for eradication?. World J. Gastroenterol..

[30] Soeung S.C., Thiep C., Duncan R., Patel M., Hennessey K. (2012). Using data to guide policy: Next steps for preventing perinatal hepatitis B virus transmission in Cambodia. Vaccine.

[31] Luman E.T., Barker L.E., McCauley M.M., Drews-Botsch C. (2005). Timeliness of childhood immunizations: A state-specific analysis. Am. J. Public Health.

[32] Kushner T., Kaplowitz E., Mei R., Xu C., Acker A., Rosenbluth E., Oredein I., Sarkar M., Terrault N., Bansa M. (2021). Adherence to pregnancy hepatitis B care guidelines in women and infants in the U.S. and evaluation of two interventions to improve care: A multi-center hospital-based study. J. Viral Hepat..

[33] Fasano M., Saracino A., Carosi G., Mazzotta F., Marino N., Sagnelli E., Gaeta G.B., Angarano G., Verucchi G., Bellissima P. (2013). Hepatitis B and immigrants: A SIMIT multicenter cross-sectional study. Infection.

[34] Sagnelli E., Sagnelli C., Pisaturo M., Macera M., Coppola N. (2014). Epidemiology of acute and chronic hepatitis B and delta over the last 5 decades in Italy. World J. Gastroenterol..

[35] Coppola N., Alessio L., Gualdieri L., Pisaturo M., Sagnelli C., Minichini C., Di Caprio G., Starace M., Onorato L., Signoriello G. (2017). Hepatitis B virus infection in undocumented immigrants and refugees in Southern Italy: Demographic, virological, and clinical features. Infect. Dis. Poverty.

[36] (2002). Global Alert and Response. Hepatitis B. Prevention and Treatment.

[37] Mele A., Tancredi F., Romanò L., Giuseppone A., Colucci M., Sangiuolo A., Lecce R., Adamo B., Tosti M.E., Taliani G. (2001). Effectiveness of hepatitis B vaccination in babies born to hepatitis B surface antigen-positive mothers in Italy. J. Infect. Dis..

[38] Calvo P.L., Pinon M., Dell’Olio D., Carpino A., Biasin E., Pizzol A., Catalano S., Peruzzi L., Rigazio C., Cisarò F. (2020). Management of Hepatitis-B Virus Infection in Immunocompromised Children. A Single Center Experience. J. Pediatr. Gastroenterol. Nutr..

[39] Indolfi G., Abdel-Hady M., Bansal S., Debray D., Smets F., Czubkowski P., van der Woerd W., Samyn M., Jahnel J., Gupte G. (2020). Management of Hepatitis B Virus Infection and Prevention of Hepatitis B Virus Reactivation in Children With Acquired Immunodeficiencies or Undergoing Immune Suppressive, Cytotoxic, or Biological Modifier Therapies. J. Pediatr. Gastroenterol. Nutr..

[40] Ghany M.G., Morgan T.R. (2020). Hepatitis C Guidance 2019 Update: American Association for the study of the Liver Diseases-Infectious Disease Society of American recommendations for Testing, Managing and Treating Hepatitis C Virus Infection. AASLD/IDSA Hepat. C Guid. Panel Hepatol..

